# Rule learning enhances structural plasticity of long-range axons in frontal cortex

**DOI:** 10.1038/ncomms10785

**Published:** 2016-03-07

**Authors:** Carolyn M. Johnson, Hannah Peckler, Lung-Hao Tai, Linda Wilbrecht

**Affiliations:** 1 UCSF Neuroscience Graduate Program, University of California San Francisco, San Francisco, California 94158, USA; 2 Department of Psychology, University of California Berkeley, Berkeley, California 94720, USA; 3 Helen Wills Neuroscience Institute, University of California Berkeley, Berkeley, California 94720, USA

## Abstract

Rules encompass cue-action-outcome associations used to guide decisions and strategies in a specific context. Subregions of the frontal cortex including the orbitofrontal cortex (OFC) and dorsomedial prefrontal cortex (dmPFC) are implicated in rule learning, although changes in structural connectivity underlying rule learning are poorly understood. We imaged OFC axonal projections to dmPFC during training in a multiple choice foraging task and used a reinforcement learning model to quantify explore–exploit strategy use and prediction error magnitude. Here we show that rule training, but not experience of reward alone, enhances OFC bouton plasticity. Baseline bouton density and gains during training correlate with rule exploitation, while bouton loss correlates with exploration and scales with the magnitude of experienced prediction errors. We conclude that rule learning sculpts frontal cortex interconnectivity and adjusts a thermostat for the explore–exploit balance.

## Abstract (web summary)

The orbitofrontal cortex is associated with foraging behaviour yet the structural changes underlying such rule-based learning remain unclear. Here, the authors imaged OFC axons throughout a digging-based odour discrimination task and found correlations between the rate of bouton turnover and the behavioural strategies of individual mice.

## Graphical abstract

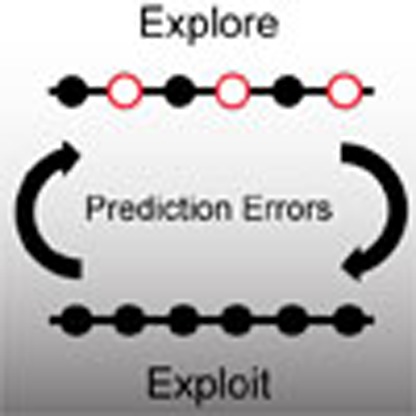

Rules are learned associations among cues, actions and outcomes used to guide actions in pursuit of a goal^1^. The prefrontal cortex is critical for the representation and implementation of rules^2^,^3^,^4^,^5^,^6^,^7^. The orbitofrontal cortex (OFC) and dorsomedial prefrontal cortex (dmPFC; including cingulate cortex and secondary motor cortex) have been proposed to encode cue–outcome and action–outcome associations, respectively^8^,^9^, and lesions of these regions impair flexible updating of choice behaviour^10^,^11^,^12^,^13^,^14^,^15^. Rearrangements of synaptic connectivity are generally thought to underlie many forms of learning and memory^16^,^17^. The *in vivo* formation and elimination of frontal cortex dendritic spines has been observed previously in response to motor learning^18^,^19^ and conditioning^20^,^21^, yet knowledge regarding the structural basis of abstract rule learning in goal directed behaviour remains limited. Here we hypothesized that rule learning—connecting cues, actions and outcomes—would drive structural plasticity in long-range axons projecting from the OFC to the dmPFC.

A fundamental problem of decision-making is the dilemma of whether to pursue a known course of action to achieve reward (exploitation) or to search alternative options in hope of a better outcome (exploration)^22^,^23^. In novel situations or under conditions of uncertainty, exploration is used to gather information on the outcomes of choices^24^,^25^. Rather than representing impulsivity or error, strategic exploration is a critical strategy for organisms to discover resources necessary for survival^26^,^27^. Strategic exploration emerges between early adolescence and adulthood in parallel with frontal cortex maturation^12^ and plays a role in formulating rules to achieve long-term goals^28^.

A balance must be struck between exploration and exploitation for optimal decision-making. Abnormalities in decision-making flexibility characterize numerous psychiatric disorders^29^. Different subregions of the frontal cortex have been implicated in exploratory and exploitative choices. The OFC and ventromedial PFC are involved in exploiting and monitoring the outcome of the current rule^8^,^30^,^31^, while the dmPFC and rostral frontal cortex may have a more specialized role in monitoring alternative strategies and exploratory actions^14^,^30^,^31^,^32^,^33^,^34^,^35^,^36^. The frontal cortex may act as a thermostat for decision-making, taking the temperature of the current environment and setting the appropriate explore/exploit strategy. We thus hypothesized that long-range connectivity from OFC to dmPFC may play a specific role in regulating exploratory versus exploitative strategy choice during rule learning.

Theories of learning have long recognized that learning is not a passive process^37^. Rather, subjects actively make predictions about the outcomes of cues and actions, and learning occurs when a subject experiences an unexpected outcome^38^. The difference between the expected result and the actual outcome is formally called a ‘prediction error'. A perfectly predicted outcome does not generate a prediction error or spur new learning, while experience of an unexpected reward generates a positive prediction error and supports responses to associated cues. An omission of an expected reward generates a negative prediction error that leads to extinction of associated behaviours. This error-driven learning figures prominently in computational models of associative learning^22^,^38^,^39^. Prediction errors adjust behaviour presumably by remodelling associations between cues, actions and outcomes. However, the neural basis of this relationship remains unclear.

Here we train mice to learn rules in a multiple choice foraging and reversal task^12^, and daily image OFC axons that project to the dmPFC. We use a reinforcement learning model^22^,^38^ to classify exploratory and exploitative strategies^30^ and compare individual differences in OFC to dmPFC connectivity with choice strategy implementation. We leverage the natural diversity of individual choice histories and resulting prediction error magnitudes to survey how prediction errors may scale plasticity at the structural level. Our data reveal a structural trace of rule learning, and illuminate neural correlates of individual differences in decision making history and strategy.

## Results

### dmPFC lesions and the explore/exploit balance

**Figure 1:**
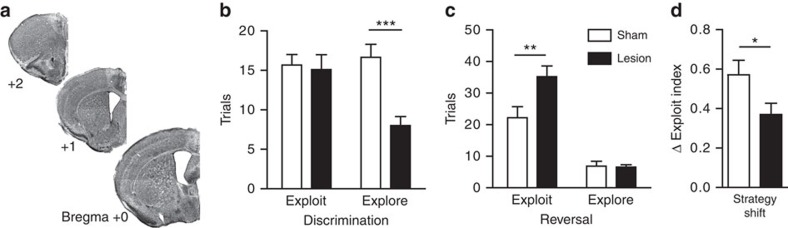
dmPFC lesions alter the explore/exploit balance. (**a**) Bilateral excitotoxic lesions of dmPFC. Cresyl-violet-stained sections show the extent of a typical lesion. (**b**) The behavioural choices were classified as explore or exploit choices using a reinforcement learning model. Lesion mice made fewer explore choices in discrimination compared with sham-operated mice (lesion: *n*=11, sham: *n*=9; strategy: *F*(1,17)=5.14, *P*=0.04; lesion: *F*(1,17)=7.34, *P*=0.02; interaction: *F*(1,17)=9.06, *P*=0.008; two-way repeated measures analysis of variance (ANOVA), Bonferroni *post hoc* test). (**c**) In reversal, lesion mice made more exploit choices (strategy: *F*(1,17)=90.66, *P*<0.0001; lesion: *F*(1,17)=5.22, *P*=0.04; interaction: *F*(1,17)=8.27, *P*=0.01; two-way repeated measures ANOVA, Bonferroni *post hoc* test). (**d**) Lesion mice shifted their strategy less from discrimination to reversal compared with sham mice, where a large number indicates more exploit choices in reversal (*t*(17)=2.19, *P*=0.04; unpaired *t*-test). ‘Exploit index'=(exploit−explore)/total trials. *P<0.05, **P<0.01, ***P<0.001. Bars represent the mean±s.e.m. Behavioural data for modelling were previously published^12^.

To first establish the role of the dmPFC in modulating exploratory and exploitative choice behaviour in mice, we used a reinforcement learning model to perform new analysis of data from a previous study of dmPFC lesions made in our lab^12^. Bilateral excitotoxic lesions were made in dmPFC in adult mice (Fig. 1a) and then mice were trained in a multiple choice foraging task. In the discrimination phase of the task, mice foraged for a buried piece of cereal reward hidden in one of four pots of scented wood shavings until reaching criterion of 8 out of 10 consecutive trials correct. The spatial location of the odour cues was shuffled on each trial. Mice freely explored the arena and indicated a choice by digging in one of the pots. In the reversal phase, the cereal reward was buried in odour 2 instead of the previously rewarded odour 1. Subjects needed to abandon the previously rewarded action, explore the alternatives and exploit the new rewarded action until again reaching criterion of 8 out of 10 trials correct.

We used a reinforcement learning model based on the Rescorla–Wagner model of expectancy driven learning^22^,^38^ (see Methods). Prediction errors are generated when the outcome does not match the expected outcome. Unexpected reward results in a positive prediction error while unexpected omission of reward results in a negative prediction error. Using animals' individual choice histories, we used the model to calculate the relative choice probabilities in each trial and estimate the magnitude of prediction errors experienced after each choice. We classified trials as exploitative if the actual choice matched the most probable choice predicted by the model and as exploratory when the choice was to any of the three less probable options^30^.

Using the model to classify choice strategy on each trial, we found that dmPFC lesion mice explored significantly less in discrimination compared with sham operated mice (Fig. 1b). In the reversal phase, lesion mice made significantly more exploitative choices (Fig. 1c). To test whether lesions impaired flexibility in choosing the appropriate strategy, we calculated the ‘exploit index' (the proportion of exploit choices) and then found the change in the ‘exploit index' between discrimination and reversal. Lesion mice tended to shift their behavioural strategy less between discrimination and reversal (Fig. 1d). In sum, mice with frontal lesions had altered strategies and inflexible responses to unexpected outcomes.

### Rule training enhances plasticity of long-range axonal projections

**Figure 2:**
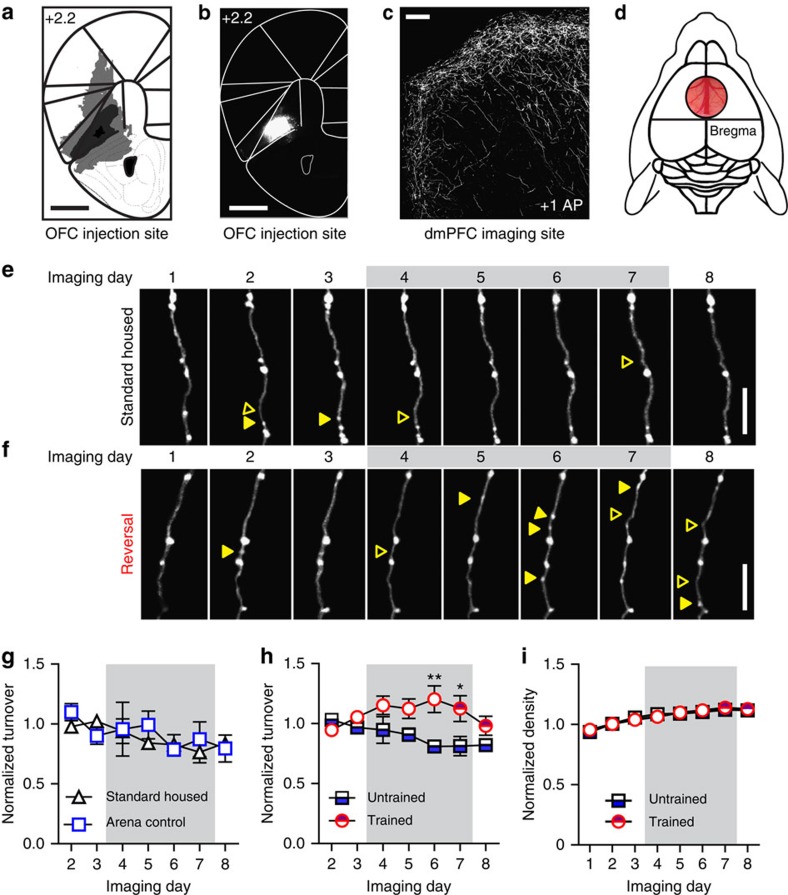
Rule training enhances OFC bouton turnover. (**a**) Schematic showing the extent of viral labelling at injection site in the OFC in all experimental mice (*n*=35). Black indicates the minimum expression and light grey is the maximum expression. Medium grey shows a representative example of labelling. (**b**,**c**) Representative image of viral labelling at the injection site in OFC (**b**) and of axonal projections to the upper layers of contralateral dmPFC (**c**). (**d**) Schematic of cranial window implant over dmPFC. (**e**,**f**) Representative *in vivo* two-photon images of axons of a ‘standard-housed' (**e**) and a ‘reversal' trained (**f**) subject across imaging sessions. Open and closed arrowheads indicate bouton gain and loss, respectively. (**g**) Bouton turnover did not differ between the ‘standard-housed' (*n*=10) and ‘arena control' (*n*=8) groups across imaging sessions (group: *F*(1,16)=0.35, *P*=0.56; time: *F*(6,96)=1.70, *P*=0.13; interaction: *F*(6,96)=0.56, *P*=0.76). (**h**) The ‘trained' group (*n*=17 mice) had significantly higher bouton turnover following the odour discrimination (day 6) and recall/reversal (day 7) sessions compared with the ‘untrained' group (*n*=18 mice; group: *F*(1,33)=14.59, *P*=0.0006; time: *F*(6,198)=0.87, *P*=0.52; interaction: *F*(6,198)=2.38, *P*=0.03). (**i**) Density did not differ between ‘untrained' and ‘trained' groups (group: *F*(1,33)=0.06, *P*=0.81; time: *F*(7,231)=63.34, *P*<0.0001; interaction: *F*(7,231)=0.88, *P*=0.52). Groups in **g**–**i** were compared using two-way repeated measures analysis of variance and Bonferroni *post hoc* tests. Data from each mouse were normalized to the average of baseline sessions and are presented as mean±s.e.m. in **g**–**i**. Scale bars, (**a**,**b**) 1 mm; (**c**) 100 μm; (**e**,**f**) 10 μm. *P<0.05, **P<0.01.

**Figure 3:**
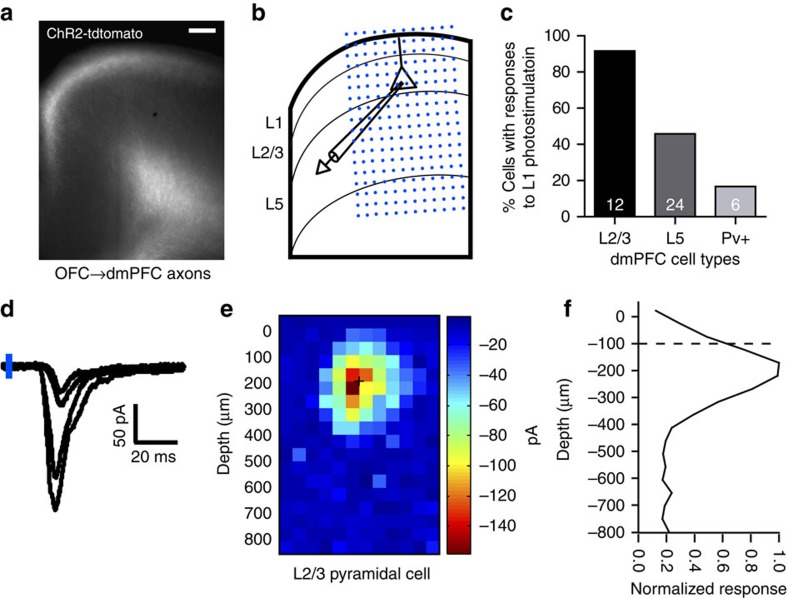
OFC axons make functional synapses onto multiple cell types in the dmPFC. (**a**) Image showing that OFC axons expressing ChR2-tdTomato project to the contralateral dmPFC. Scale bar, 100 μm. (**b**) We used photostimulation of OFC axon terminals and whole-cell recording in dmPFC to confirm functional connectivity. A 12 × 18 photostimulation grid (50-μm spacing) was used to assess laminar input patterns. (**c**) Proportion of cell types in dmPFC with current responses to photostimulation of L1 OFC axons. Cell types include L2/3 and L5 pyramidal cells identified by morphology and distance from the surface, and parvalbumin-positive (Pv+) putative interneurons identified in *Pvalb-IRES-Cre* mice. Number insets are the number of cells recorded. (**d**) Example current traces from axon terminal photostimulation pulses at different locations in the grid. (**e**) Heat map of current responses in a L2/3 pyramidal cell to photostimulation of OFC axons from the contralateral hemisphere. Note that the first row is outside of the slice. + Indicates location of the soma. (**f**) Quantification of the responses in **e** by depth from the surface. Responses were normalized to the maximum response. OFC axons in L1 (indicated by dashed line) are capable of driving substantial responses in the soma.

Our next goal was to study inputs to the dmPFC from the OFC subregion of frontal cortex. We used adeno-associated virus to label OFC axons that project to the contralateral dmPFC (Fig. 2a-c). To confirm that OFC axons make functional synapses in the dmPFC, we virally expressed the gene encoding channelrhodopsin (ChR2) in OFC axons (Fig. 3a). We then used laser scanning photostimulation of OFC axon terminals containing ChR2 combined with whole-cell recording of downstream cells to confirm monosynaptic connectivity (Fig. 3b–f). OFC axons in L1 made functional connections with 92% of L2/3 pyramidal cells (11 out of 12 cells), as well as L5 pyramidal cells and parvalbumin expressing putative interneurons (Fig. 3c).

We next moved to our core experiment, using adeno-associated virus to deliver the gene encoding green fluorescent protein (EGFP) to OFC neurons and imaging OFC→dmPFC projection axons using two-photon microscopy. To image fluorescently labelled OFC axons in the living brain, we implanted a chronic cranial window over the dmPFC (Fig. 2d). After a recovery period, segments of OFC axons were imaged in the dmPFC daily for a baseline period of 3 days in the afternoon. Mice were then exposed to control conditions or trained in the foraging task in the morning and the OFC→dmPFC axons continued to be imaged daily in the afternoon (Table 1; Supplementary Fig. 1). We tracked gains and losses of boutons using established detection thresholds^40^,^41^ (see Methods; Fig. 2e, f) blind to treatment group.

Our goal was to document structural remodelling in OFC axons that might occur in response to control conditions or rule learning. Our experimental design included multiple control groups to test whether factors independent from rule learning were sufficient to drive OFC→dmPFC bouton plasticity. The ‘untrained' control conditions included ‘standard-housed' mice (*n*=10 mice), which were simply imaged daily, and a yoked ‘arena control' group (*n*=8 mice), which experienced the same handling and cues as ‘trained' mice, but without the experience of reward (Table 1; Supplementary Fig. 1). Following 3 days of baseline imaging, ‘trained' mice underwent habituation to the arena on day 4 and shaping to learn to dig for buried piece of cereal on day 5. On day 6, ‘trained' mice completed the odour discrimination phase, learning that one of four odour choices contained a buried reward. On day 7, the ‘trained' group diverged into two conditions. Both groups completed a recall to criterion of the odour discrimination from the previous day, whereupon the ‘recall-only' group (*n*=8) was returned to the homecage. The ‘reversal' group (*n*=9) completed a reversal to criterion in the same session as the recall, with the reward now buried in odour 2. On day 8, all mice stayed in the homecage and were imaged for a final session.

Comparing the ‘untrained' (*n*=18) and ‘trained' (*n*=17) groups, we found that rule training in the multiple choice foraging task significantly enhanced OFC→dmPFC bouton turnover (gain+loss/2 × total boutons) on both day 6 (discrimination) and day 7 (recall/reversal; Fig. 2h). Bouton density was not different between groups across training (Fig. 2i). After training, turnover returned to baseline level in the final imaging session, indicating that experience-dependent changes were likely captured in the same-day imaging sessions. Thus, multiple choice rule learning encompassing complex cue–action–outcome associations drove significant increases in OFC→dmPFC plasticity.

**Figure 4:**
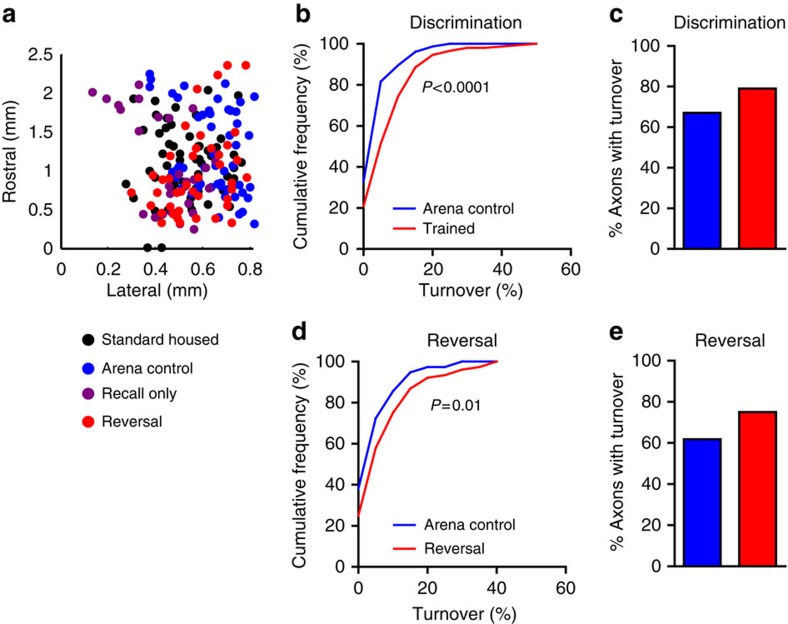
Recruitment of plasticity in the majority of axons in discrimination and reversal learning. (**a**) Locations of imaged axons in dmPFC. (**b**) Cumulative frequency histogram of the percentage of total boutons gained and lost on day 6 in ‘arena control' (*n*=76 axons) and odour discrimination ‘trained' (*n*=148 axons) mice. The distribution was shifted significantly towards higher turnover in ‘trained' group (KS statistic=0.47, *P*<0.0001). (**c**) ‘Trained' mice had more axons (79.5%) with some level of bouton turnover compared with ‘arena control' mice (67.1%). (**d**) On day 7, the cumulative frequency distribution of turnover was shifted towards higher turnover in the ‘reversal' group (*n*=76 axons) compared with ‘arena control' group (*n*=76 axons; KS statistic=0.25, *P*=0.01). (**e**) ‘Reversal' trained mice had more axons that gained or lost boutons (75.0%) than ‘arena control' mice (61.9%).

To test whether plasticity was a general phenomenon or restricted to a smaller proportion of ‘hot' axons, we compared cumulative frequency histograms of bouton turnover from all axons imaged (323 axons from 35 mice; Fig. 4a). On discrimination day 6, axons from the ‘trained' group showed higher bouton turnover compared with axons from the ‘arena control' group (Fig. 4b). The large majority of axons in ‘trained' mice (80%) showed plasticity, with ∼13% more axons recruited to active turnover compared with controls (Fig. 4c). On recall/reversal day 7, axons in the ‘reversal' group again showed higher bouton turnover compared with axons from ‘arena control' mice (Fig. 4d), with again ∼13% more axons showing turnover in the ‘reversal' group (Fig. 4e).

### Specificity of plasticity in OFC projections to dmPFC

To exclude the possibility that enhanced bouton turnover was simply a result of environmental enrichment, we compared bouton plasticity between the ‘standard-housed' and ‘arena control' groups. The ‘arena control' group experienced the same environmental context and cues as the ‘trained' mice, but without the experience of reward (Supplementary Fig. 1). Bouton turnover was not significantly different between ‘standard-housed' and ‘arena control' groups across days (Fig. 2g).

**Figure 5:**
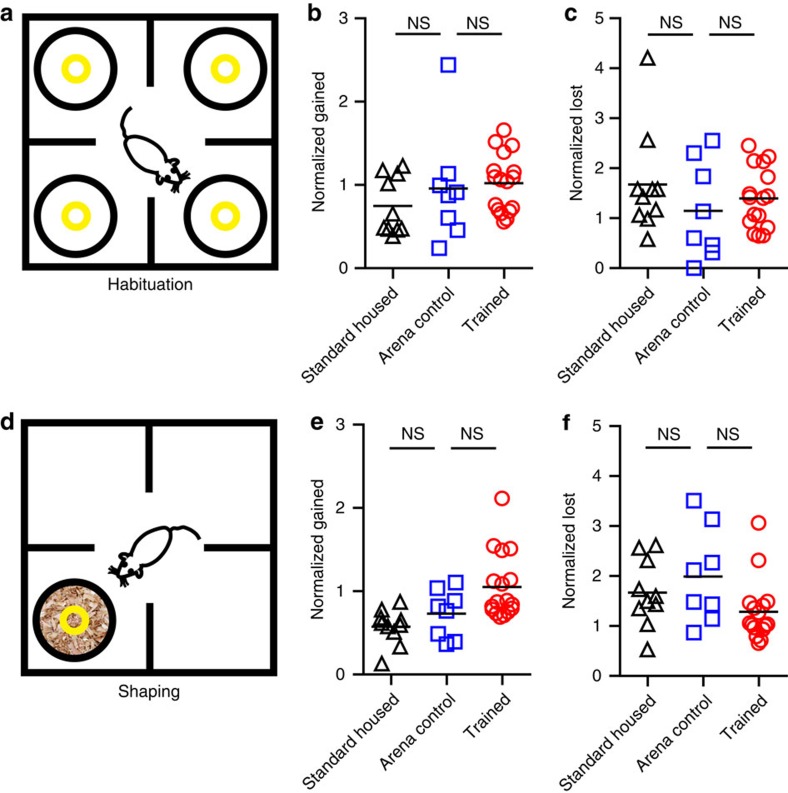
Environmental enrichment, novel food reward and simple instrumental learning were not sufficient to drive axon plasticity above control levels. (**a**) Schematic of behaviour arena on habituation day 4. Pots placed in the corners of the arena were baited 12 times with food rewards at pseudorandom intervals within a 30-min session, uncoupled from the animals' behaviour. (**b**) Bouton gains normalized to the average baseline level for each mouse did not differ among the ‘standard-housed' (*n*=10), ‘arena control' (*n*=8) or ‘trained' (*n*=17) groups (*F*(2, 32)=1.23, *P*=0.31). (**c**) Normalized bouton losses did not differ among groups (*F*(2, 31)=0.88, *P*=0.42). One outlier from the ‘trained' group was excluded from analysis of normalized lost (3.71 s.d.'s from the mean). (**d**) Schematic of behaviour arena on shaping day 5. Mice learned to dig in increasing levels of wood shavings to find a buried cereal reward. Shaping consisted of 12 trials and the location of the pot was changed each trial. (**e**) Normalized bouton gains were not significantly different (*F*(2, 32)=7.13, *P*=0.003, Bonferroni *post hoc* tests >0.05). (**f**) There were no significant differences in normalized bouton losses (*F*(2, 31)=2.73, *P*=0.08). One outlier from the ‘trained' group was excluded from analysis of normalized loss (3.65 s.d.'s from the mean). Each symbol represents one mouse. Graphs show mean±s.e.m. Groups are compared using one-way analysis of variance with Bonferroni *post hoc* test.

We next examined the possibility that increased bouton dynamics were due to the experience of reward, independent from learning rules. On day 4 (habituation), mice in the ‘trained' group received rewards at pseudorandom intervals in the training arena, uncoupled from the animals' behaviour (Fig. 5a). We found no differences in bouton gain (Fig. 5b) or loss (Fig. 5c) among groups on day 4. We also found no significant difference comparing between baseline and day 4 turnover within the ‘trained' group (*P*=0.08).

On day 5, mice were shaped to dig for a piece of cereal buried under wood shavings in a single pot (Fig. 5d). We found a non-significant trend towards higher bouton gain in the ‘trained' group (Fig. 5e). There were no differences found in bouton loss (Fig. 5f). In comparing baseline with day 5 turnover within the ‘trained' group, we found no significant difference (*P*=0.26). Together, these control conditions establish that novel sensory cues, non-contingent rewards and the simple instrumental action of digging for reward with a single choice did not substantially enhance structural plasticity in OFC→dmPFC axons.

To test whether the experience-dependent plasticity observed following rule training on day 6 was specific to the OFC→dmPFC projection, we also imaged OFC projections to primary motor cortex (M1). The OFC→M1 axons showed no difference in turnover between ‘untrained' and ‘trained' mice on day 6 (Supplementary Fig. 2), suggesting pathway-specific plasticity within the OFC projection to dmPFC in rule learning.

### OFC to dmPFC connectivity and the explore/exploit balance

**Figure 6:**
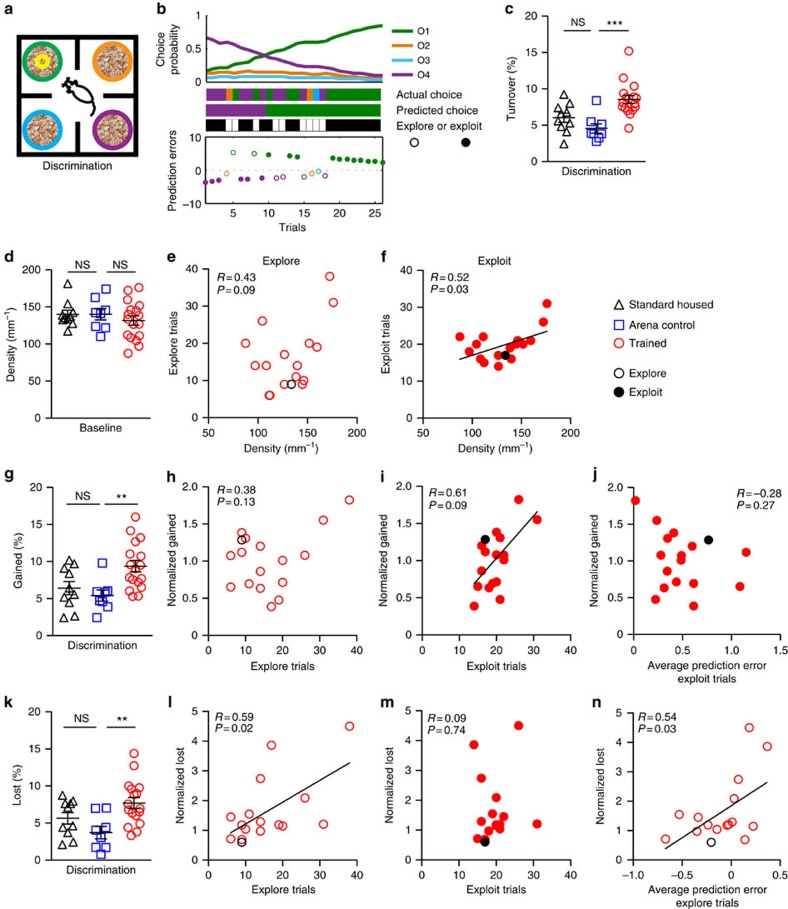
Bouton dynamics following multiple choice discrimination learning correlate with strategy and scale with prediction errors. (**a**) Schematic of day 6 odour discrimination arena. (**b**) Computational model of choice probabilities and prediction errors in an example subject. If the actual choice (top bar) matched the most probable choice predicted by the model (middle bar), then trials were classified as exploitative (black bottom bar; closed circles). Choice of any of the three alternative choices was classified as exploratory (white bottom bar; open circles). The innate odour preferences across all mice in the first four trials set the initial choice probabilities. ‘O4' was initially preferred by all mice, and through trial and error the rewarded odour ‘O1' gained value and probability of being chosen. In subsequent panels, the black circles refer to this example subject. (**c**) Bouton turnover on day 6 was increased in the ‘trained' group (*n*=17 mice) compared with ‘standard-housed' (*n*=10) and ‘arena control' (*n*=8) groups (*F*(2,32)=10.81, *P*=0.0003). (**d**) Baseline bouton density did not differ among groups (*F*(2,32)=0.63, *P*=0.53). (**e**,**f**) Bouton density did not predict the number of explore choices (**e**), but did predict exploit choices (**f**). (**g**) The percentage of total boutons gained was enhanced in the ‘trained' group after discrimination learning (*F*(2,32)=6.36, *P*=0.005). (**h**,**i**) Baseline normalized bouton gain in ‘trained' mice correlated with the number of exploit choices (**i**), but not explore choices (**h**). (**j**) Average prediction errors from exploit trials did not correlate with bouton gain. (**k**) The percentage of total boutons lost was enhanced in the ‘trained' group (*F*(2,32)=6.06, *P*=0.006). (**l**,**m**) Baseline normalized loss was correlated with the number of explore trials (**l**), but not exploit trials (**m**). (**n**) The average prediction errors from exploratory trials correlated with bouton loss. One outlier with low baseline loss was excluded from the analysis of normalized losses (**l**–**n**; 3.53 s.d.'s from the mean; see Supplementary Fig. 3). Each symbol represents a mouse. Data in (**c**,**d**,**g**,**k**) are mean±s.e.m., and are compared using one-way analysis of variance with Bonferroni *post hoc* tests. Correlations are Pearson's correlation coefficient. **P<0.01, ***P<0.001.

**Figure 7:**
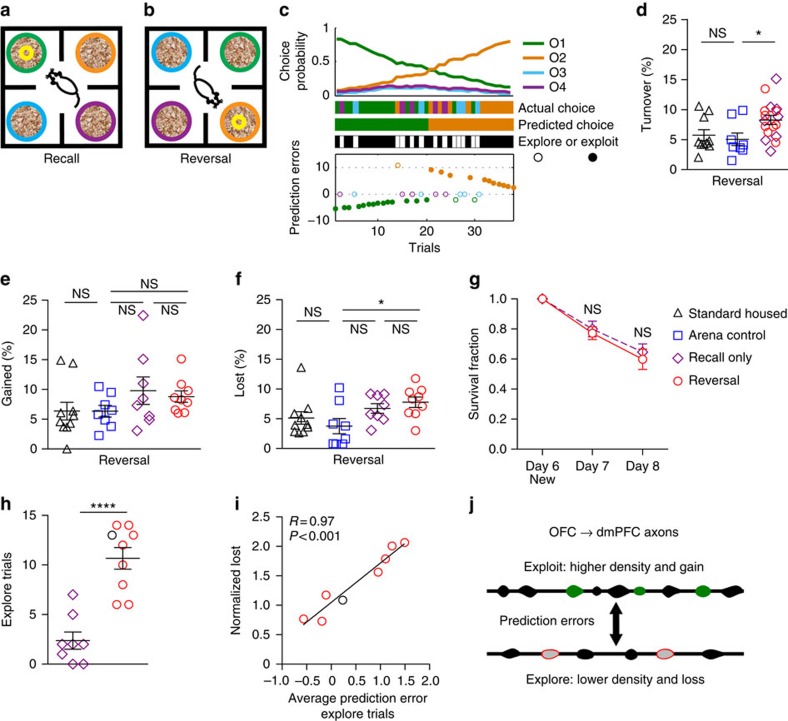
Bouton loss following reversal learning scales with exploratory prediction errors. (**a**,**b**) Schematic of day 7 recall (**a**) and reversal (**b**). (**c**) Model of choice probability and estimated prediction errors for explore/exploit trial classification in the reversal phase. Black circles in **g** and **h** indicate the representative mouse shown in **c**. (**d**) Bouton turnover was enhanced in the ‘trained' group (*n*=17) on day 7 compared with ‘standard-housed' (*n*=10) and ‘arena control' (*n*=8) groups (*F*(2,32)=4.00, *P*=0.03; one-way analysis of variance (ANOVA) with Bonferroni *post hoc* tests). (**e**) Comparing among all groups, there were no differences in bouton gain (*F*(3,31)=1.31, *P*=0.29; one-way ANOVA with Bonferroni *post hoc* tests). (**f**) The ‘reversal' group lost significantly more boutons than the ‘arena control' group (*F*(3,31)=2.95, *P*=0.048; one-way ANOVA with Bonferroni *post hoc* tests). (**g**) The survival fraction of boutons gained on day 6 to subsequent imaging sessions was not significantly different between the ‘recall-only' (*n*=8) and ‘reversal' group (*n*=9; group: *F*(1,15)=0.27, *P*=0.61; time: *F*(2,30)=53.99, *P*<0.0001; interaction: *F*(2,30)=0.22, *P*=0.81; repeated measures ANOVA with Bonferroni *post hoc* tests). (**h**) The ‘reversal' group made more exploratory choices than the ‘recall-only' group (*t*(15)=5.84, *P*<0.0001; unpaired *t*-test). (**i**) Average prediction errors on exploratory trials correlated with normalized bouton loss (Pearson's correlation). One outlier was excluded from **i** (2.21 s.d.'s from mean; see Supplementary Fig. 3). (**j**) Summary schematic. Each symbol represents a mouse. Data in (**d**–**h**) show mean±s.e.m. * *P*<0.05, **** *P*<0.0001.

After establishing that rule training altered OFC→dmPFC bouton turnover, our next goal was to investigate whether bouton plasticity reflected each individual's strategy and feedback experience during rule learning on day 6 and 7. Using individual animals' unique choice histories, we used the model to calculate the relative choice probabilities on each trial and estimated the magnitude of prediction errors experienced after each choice (Figs 6b and 7c). As described previously, we classified choices as exploitative if the actual choice matched the most probable choice predicted by the model and as exploratory if any of the three less probable options was chosen^30^. Both strategies can be either rewarded or unrewarded. Note on day 6 that most logical (probable) choice based on the evidence gathered switches from ‘O4' (unrewarded) to ‘O1' (rewarded; Fig. 6b), and from ‘O1' (unrewarded) to ‘O2' (rewarded) in reversal on day 7 (Fig. 7c). While it may be tempting to conflate exploration with error, it may also serve as a means of hypothesis testing. For example, the representative subject in Fig. 6b methodically samples each choice in turn in an exploratory bout late in the session before going on a rewarded streak until the end of the session.

Comparing ‘trained' and ‘standard-housed' groups to the ‘arena control' group on discrimination day 6, we observed a nearly twofold increase in OFC→dmPFC bouton turnover in ‘trained' mice (Fig. 6a,c). The increase in bouton turnover in the ‘trained' group was driven by greater percentages of total boutons both gained (Fig. 6g) and lost (Fig. 6k) from the previous day. Groups showed comparable mean baseline OFC→dmPFC bouton density (Fig. 6d). Exploitative choices on discrimination day 6 were positively correlated with both baseline bouton density (Fig. 6f) and subsequent gains of new boutons (Fig. 6i), but not with bouton loss (Fig. 6m). In contrast, exploratory choices were correlated with bouton loss (Fig. 6l), but not with baseline density (Fig. 6e) or bouton gain (Fig. 6h). We did not find correlations between OFC bouton plasticity and either the total number of rewards received or the number of trials to reach criterion (Table 2), indicating that this was unlikely to be a simple reward or practice effect.

We next tested whether bouton gain and loss on day 6 were scaled by individual differences in prediction errors. The average prediction error individually experienced on exploit trials was not correlated with OFC bouton gain (Fig. 6j). However, the average prediction errors generated by exploration trials were correlated with OFC bouton loss (Fig. 6n; see also Supplementary Fig. 3), such that greater OFC bouton losses occurred when outcomes from exploration yielded better than expected outcomes (positive prediction errors). Together, these results suggest that the strength of connectivity between the OFC and dmPFC regulates the explore/exploit balance, and that prediction errors may dynamically update this connectivity (Fig. 7j).

**Figure 8:**
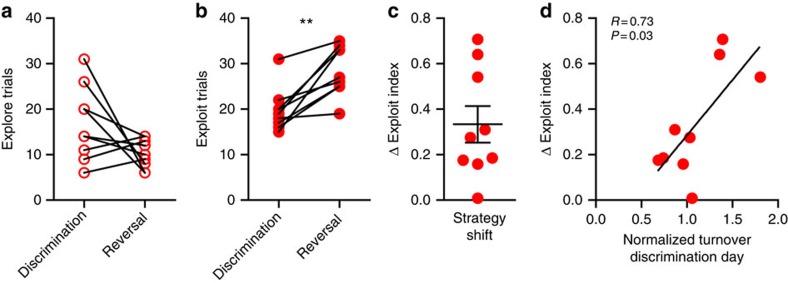
Bouton plasticity precedes updating of decision-making strategy. (**a**) Mice (*n*=9) explored less in reversal compared with discrimination (*t*(8)=1.91, *P*=0.09; paired *t*-test). (**b**) Mice exploited significantly more in reversal (*t*(8)=4.70, *P*=0.002; paired *t*-test). (**c**) This shift in strategic choice is captured by the change in the ‘exploit index' (exploit−explore/total trials), where a positive number indicates proportionally more exploitation in reversal. (**d**) The amount of bouton turnover observed following discrimination training on day 6 correlated with the shift in the ‘exploit index' on day 7 (Pearson's correlation coefficient). Bouton plasticity was related to flexibility in decision-making strategy. Connected symbols are the same mouse on different days. Graph (**c**) shows mean±s.e.m. ** *P*<0.01.

On the seventh day of training, mice were tested for recall of the previous day's rule (Fig. 7a), which was then reversed within the same session for the ‘reversal' group (Fig. 7b). During recall, mice required fewer trials to reach criterion than during initial acquisition (discrimination: 37.41±3.15; recall: 10.53±0.66 trials; *t*(16)=7.75, *P*<0.0001), indicating >24 h memory of the odour-reward contingencies. Mice showed a tendency to explore less in reversal compared with discrimination (Fig. 8a). Mice also made significantly more exploitative choices (Fig. 8b), likely reflecting decreased uncertainty with experience in the task. We quantified this shift in strategy as the change in the ‘exploit index' between sessions (Fig. 8c). Within the OFC→dmPFC projection, we found that the magnitude of bouton turnover on day 6 predicted the strategy shift on day 7 (Fig. 8d). Connecting the fact that enhanced bouton plasticity is correlated with behavioural flexibility, and the fact that mice with dmPFC lesions showed smaller experience-dependent changes in behavioural strategy (Fig. 1d), we suggest the shift in strategy may be at least partially dependent on plasticity of synaptic connections made in the dmPFC.

### Reversal training drives exploration and bouton loss

Analysis of OFC→dmPFC bouton plasticity on day 7 showed ‘trained' mice had higher turnover than controls (Fig. 7d). Breaking turnover down into the percentage of boutons gained and lost on day 7, we observed that the ‘reversal' group lost significantly more boutons (Fig. 7f) compared with ‘arena control' group, but found no differences in bouton gain (Fig. 7e). When comparing the ‘recall-only' and ‘reversal' groups, we found no differences in bouton gain, loss or the persistence of new boutons gained after odour discrimination training (Fig. 7g), indicating that reversal is likely new learning rather than erasure of previous associations^42^. ‘Reversal' group mice showed a significant increase in the number of exploratory trials compared to the ‘recall-only' group (Fig. 7h). On day 7, bouton loss scaled with average prediction errors from exploratory trials (Fig. 7i; Supplementary Fig. 3), replicating observations from day 6 in which greater bouton loss was observed following more positive outcomes from exploration. Note that the magnitudes of predication errors have a larger range on day 7 (Fig. 7i) compared with day 6 (Fig. 6n). Increased experience with the task creates stronger expectations, which when violated in reversal generate larger prediction errors. Together, the results from day 6 and 7 show that behavioural conditions that encourage strategic exploration, and positive outcomes from this exploration, promote pruning in the projection from OFC to dmPFC (Fig. 7j).

## Discussion

To study how rule learning impacts frontal cortex connectivity and plasticity, we used *in vivo* two-photon imaging to observe long-range OFC→dmPFC projection axons before and after training in a multiple choice foraging task. We discovered that rule learning resulted in both enhanced bouton gain and loss. Further parsing these findings, prior bouton density and subsequent new gains following discrimination training were related to learning and exploiting a rule. Conversely, bouton loss was correlated with greater strategic exploration and was scaled by the magnitude of prediction errors generated by exploration. These results provide new, structural evidence that complex associations among cues, actions and outcomes are stored in the brain at the synaptic level. These data are complementary to previous *in vivo* electrophysiology studies showing that neurons in the rodent, primate and human prefrontal cortex encode abstract task rules in a context-dependent manner^2^,^3^,^4^,^5^,^6^,^7^,^43^. The neural representation of rules can be stable across days^6^ indicating that lasting changes to the network support memory and recall of rules. Our imaging data ‘connect the dots' by documenting changes in connectivity among neurons that occur during task learning and extend our understanding by identifying structural changes that correlate with specific aspects of rule learning strategy and experience.

Our data suggest the remodelling of OFC→dmPFC axons during rule training was widespread, rather than concentrated in a few ‘hot' axons. A large cranial window allowed imaging access to axons projecting throughout the upper layers of a 2-mm^2^ region of dmPFC. Here ∼80% of sampled axons showed experience-dependent plasticity during rule training (Fig. 4c). From our imaging data, we can only indirectly infer changes in the number of synapses. However, *in vivo* bouton intensity has previously been shown to correlate with volume, number of synaptic vesicles, size of the PSD, and number of synapses^44^,^45^. OFC axons made functional synapses with nearly all L2/3 pyramidal cells sampled (Fig. 3c). These structural plasticity data can be interpreted to support models in which the prefrontal cortex can efficiently encode decision variables by making small changes to the activity of a broad population of neurons, rather than making focal changes to a few highly selective neurons^46^,^47^.

Increasingly, it is understood that flexible rule-guided behaviour is the product of interactions among multiple brain regions^31^,^48^. We used targeted viral labelling to isolate the synaptic intersection between two critical frontal cortex hubs for flexible behaviour, the OFC and dmPFC. We found that the pretraining strength of the connection between these hubs, measured by bouton density, was predictive of exploitative choice strategy during odour discrimination training (Fig. 6f). Experience with the task further sculpted this connectivity through bouton gain and loss (Fig. 6g,k), preceding adaptations in subsequent choice strategy (Fig. 8d). Lesions of the dmPFC reduce behavioural flexibility^12^ and shifts in strategy in our task (Fig. 1d), lending support to the idea that plasticity of synaptic connections in this region is essential for adaptive behaviour. Comparing OFC projections to M1 and to dmPFC, we found pathway-specific plasticity in dmPFC during learning (Supplementary Fig. 1). Rule learning selectively alters the flow of information from the OFC to the dmPFC, with experience toggling the pipeline via bouton gain and loss (Fig. 7j).

We observed significantly enhanced bouton plasticity following discrimination and recall/reversal training (Figs 2h, 6c and 7d), but not following control experiments of enrichment (Fig. 2g), reward experience (day 4; Fig. 5a–c), or simple instrumental learning (day 5; Fig. 5d–f). We also found that bouton turnover with discrimination and recall/reversal training did not correlate with the number of rewards or trials completed (Table 2), suggesting that our findings cannot be attributed solely to reward or practice effects. The return of turnover to baseline levels on day 8 when no training occurs suggests that plasticity effects are captured by imaging the same day as training and do not extend to subsequent days. Our results are consistent with electrophysiology studies showing that neurons in the dmPFC respond preferentially to combinations of decision variables such as choice and outcome^49^,^50^. Here we used a reinforcement learning model to classify trial-by-trial choice strategies and found the interaction between strategy and outcome (prediction error magnitude for explore trials) correlated with bouton loss (Figs 6n and 7i).

Proponents of the ‘Bayesian brain hypothesis' argue that the brain is essentially a prediction machine that seeks to build accurate internal models of the environment^51^,^52^. Incoming information is compared against this internal model and a mismatch creates a prediction error signal that can be used to update the model. There is evidence of predictive coding observed from neural firing rates in sensory systems^53^,^54^, sensorimotor cortex^55^ and in regions involved in decision-making^42^,^56^,^57^,^58^. In this paper, we put forth the hypothesis that prediction errors update internal models by altering the structural connectivity among neurons. We support this hypothesis with novel data showing that bouton plasticity is scaled by prediction error magnitude. Our experiments provide a potential mechanism for the theoretical framework of prediction error-driven learning. Future studies may use our observations as a template to study the structural basis of prediction error updating in other neural systems.

Our experiments indicate that OFC→dmPFC plasticity is scaled by unexpected feedback and correlates with subsequent shifts in strategy. We can extrapolate from this finding that the accumulation of life experiences may result in different neural connectivity and decision-making patterns. A prolonged history of predictable rewarding outcomes may strengthen OFC→dmPFC connectivity and dampen the exploratory spirit. Meanwhile, surprising positive experiences after exploration may disengage OFC→dmPFC connectivity and encourage further exploration and risk-taking. For optimal decision-making, subjects need to be able to toggle between exploration and exploitation in response to current environmental conditions and internal motivational states. Exploration is beneficial to gather information in novel or dynamic environments, while exploitation strategy will yield more rewards in stable conditions. Tipping the balance of strategy towards either extreme may result in psychiatric illness such as addiction. We propose that the OFC→dmPFC circuit is tuned by experience to set the temperature of decision-making strategy. It will be interesting to see if these results are replicated in human functional connectivity studies and if therapeutic interventions that alter human behaviour also alter connectivity in this circuit.

In our study, we found that bouton turnover was enhanced with both rule acquisition during the odour discrimination on day 6 and recall/reversal training on day 7 (Fig. 2h). The significant plasticity observed on day 6 may be interpreted as surprising in light of older studies that find OFC lesion or inactivation can significantly impair reversal learning but has no effect on acquisition of a discrimination task^11^,^59^,^60^. However, dmPFC lesions and inactivations have been shown to accelerate task acquisition in previous studies^61^,^62^ and in our own data^12^. In our multiple choice task, dmPFC lesions reduced exploratory choices and hastened completion of the discrimination phase (Fig. 1b). We observed a non-significant trend towards increased gains following shaping on day 5 (Fig. 5e). Shaping could be considered a simplistic form of rule learning (association of environmental cues with the action of digging and reward), although with the critical distinction that it does not involve choice among competing alternatives^1^. We conclude that the role of the OFC and dmPFC in discrimination learning may be more evident in tasks with multiple choices and where exploration of alternatives becomes a relevant variable.

Another potentially surprising and informative detail of our data is that boutons gained during discrimination (on day 6) were not preferentially lost when the rule was reversed (on day 7; Fig. 7g). Instead, we observed that most boutons gained with rule training in the discrimination phase persisted through the end of training. Our data are in line with numerous studies showing that experience results in new persistent synaptic structures^18^,^19^,^21^,^63^,^64^,^65^. These results contrast with the finding that spines in frontal cortex are eliminated and formed at the same dendritic location during fear extinction and reconditioning, respectively^20^. One interpretation of our data is that new boutons leave a lasting structural trace of a rule that can be reactivated when contextually appropriate^66^. This interpretation is consistent with theories that posit the OFC encodes a cognitive map of task space, which allows animals to flexibly apply a variety of stored rules depending on the current context^42^.

In sum, our observations support the hypothesis that enhanced plasticity of OFC→dmPFC boutons that occurs with multiple choice training represents a structural trace of rule learning, above and beyond the simpler experiences of reward, enrichment or total trial number. This plasticity was long lasting and was observed on the majority of axons sampled. We used a reinforcement learning model to classify choice strategies and quantify prediction error magnitudes based on the unique choice histories. Further analysis of correlates of OFC→dmPFC connectivity showed a bidirectional relationship with choice strategy. OFC→dmPFC bouton density before training and experience-dependent bouton gain showed a relationship with exploitative choices, while losses were associated with greater exploration. Losses also scaled with individual experience of exploratory prediction errors, potentially representing learning from outcome feedback. We conclude that both a history of strategy and a history of feedback experience sculpt frontal circuits at the level of axonal boutons and may adjust a thermostat for future decision-making.

## Methods

### Animals

Male C57Bl/6J *Mus musculus* (2–3 months old) were group housed 2–5 per cage on a 12/12 reverse light cycle (lights off at 10 a.m.). Experiments took place during the dark period. All animals received nesting material and plastic hut in their homecage. Littermates were matched across experimental groups. Animals were food deprived to 90% of free feeding weight 3 days before and during behavioural experiments. All procedures were approved by the Ernest Gallo Clinic and Research Center and UC Berkeley Animal Care and Use Committees.

### Surgery

All surgical procedures were performed under isoflurane anaesthesia. Viral labelling techniques were used to identify projections from the OFC to the dorsomedial frontal cortex. Using a Nanojet II injector (Drummond Scientific Company, Broomall, PA), 50 nl of AAV2/1-CAG-eGFP (Addgene plasmid 28014; UNC Vector Core) or 500 nl of AAV2/1-CAG-ChR2-tdTomato (UNC Vector Core) was injected to the left OFC (anterior 2.3, lateral −1.7 and ventral 2.5) 2–6 weeks before experiments (Fig. 2a). In a separate procedure, a ∼3-mm craniotomy was made over the dorsomedial frontal cortex of both hemispheres, and sealed with a glass coverslip (Fig. 2d) as described previously^40^. The craniotomy was placed immediately rostral to bregma. Mice were allowed to recover for 7–14 days before imaging.

### Channelrhodopsin assisted circuit mapping of OFC inputs to dmPFC

In a separate set of mice, we injected 500 nl of AAV2/1-CAG-ChR2-tdTomato (UNC Vector Core) in the left OFC and allowed 1 month of expression prior to making slices in adult 2–3-month-old male mice (Fig. 3a). Animals were anaesthetized with a ketamine–xylazine mixture (70 mg kg^−1^ ketamine and 10 mg kg^−1^ xylaxine) and perfused through the heart with ice-cold artificial cerebrospinal fluid (ACSF) (in mM; 120 NaCl, 2.5 KCl, 26.2 NaHCO_3_, 1.25 NaH_2_PO_4_, 11 glucose, 2.5 CaCl_2_ and 1.3 MgCl_2_) aerated with 95% O_2_ and 5% CO_2_. The brain was rapidly removed and placed in ice-cold cutting buffer bubbled with 95% O_2_ and 5% CO_2_ (2.5 KCl, 25 NaHCO_3_, 1.25 NaH_2_PO_4_, 25 D-glucose, 0.5 CaCl_2_, 7 MgCl_2_, 110 choline-Cl, 3 mM Na-pyruvate and 11.6 Na-ascorbate). Coronal slices (300 μm) of the dmPFC were cut on a vibratome and incubated in oxygenated ACSF for 1 h at 37 °C and 30 min at room temperature before recordings.

Neurons (*n*=42 cells from 13 mice) in the contralateral dmPFC were patched with borosilicate pipettes (4–6 MΩ; internal solution: 110 Cs-methanesulfonate, 5 NaCl, 10 BAPTA, 10 HEPES, 2 MgCl_2_, 10 Na_2_-phospocreatine, 4 ATP-Na^+^, 0.3 GTP-Na^+^ and 0.1% biocytin). L2/3 and L5 pyramidal cells were identified by morphology and distance from surface. Parvalbumin-positive putative interneurons were identified in *Pvalb-IRES-Cre* mice (B6;129P2-*Pvalb*^*tm1(cre)Arbr*^/J; stock number 008069 from Jackson Labs) crossed with Ai14 reporter mice (B6;129S6-*Gt(ROSA)26Sor*^*tm14(CAG-tdTomato)Hze*^/J; stock number 007908 from Jackson Labs). Recordings were performed at 32 °C in ACSF with 0.5 μM TTX (Tocris Bioscience) and 100 μM 4-AP (Sigma) to block action potentials and restore presynaptic glutamate release, respectively. Photostimulation was with a blue laser (473 nm). Beam position was controlled with galvanometers and steered through an air immersion objective (4x magnification 0.16 numerical aperture; UPlanSApo, Olympus). For each recorded cell, laser power was adjusted to yield peak excitatory post-synaptic current (EPSC) amplitudes of ∼100 pA. We delivered light pulses (1-ms duration; interstimulus interval 800 ms) on a 12 × 18 grid (50-μm spacing) in a pseudorandomized pattern to avoid sequence-specific responses. Mapping experiments were repeated three to five times for each cell and presented as average responses. EPSCs were recorded in voltage clamp (−70 mV) and excluded if access resistance exceeded 30 MΩ (MultiClamp 700B amplifier and pClamp 10 data acquisition software, Molecular Devices).

### Four-choice odour discrimination and reversal task

The training arena was a clear acrylic box (12′′ × 12′′ × 9′′), with four internal walls (3′′) to partially divide the arena into four quadrants. White ceramic pots were used to present odour stimuli (2.9′′ diameter and 1.75′′ deep). A clear acrylic cylinder (6′′ diameter) was used to confine the mouse in the centre of the maze between trials while pots were baited and rearranged. Food rewards were ∼10-mg pieces of Honey Nut Cheerios (General Mills, MN). Odour cues were scented wood shavings (anise extract; McCormick, Hunt Valley, MD; clove, litsea, and eucalyptus oils ; San Francisco Massage Supply Company, San Francisco, CA; thymol, Alfa Aesar, Ward Hill, MA). On days 6 and 7, all pots were sham baited with a piece of cereal secured under a mesh screen.

Training in the task proceeded as previously described^12^. Mice were trained in the morning, and imaged 2–6 h later. Following 3 days of baseline imaging, mice were habituated to the arena on day 4 (Fig. 5a). Four empty pots were placed in each quadrant of the arena, and baited with cereal pieces every 10 min, for a total of 30 min. On day 5, mice were shaped to dig in wood shavings (Hartz Mountain Corporation, Secaucus, NJ) to obtain a buried food reward (Fig. 5d). Only one pot was used, and the food reward was covered with increasing amounts of wood shavings across 12 untimed trials. The pot quadrant location was changed each trial. Mice were returned to the central start cylinder between trials. Mice rapidly learned to uncover the buried food, and shaping typically was completed within ∼1 h. Note that the same total number of food rewards (12) was delivered on day 4 and 5.

On day 6, mice learned to discriminate among four pots with different scented wood shavings (anise, clove, litsea and thyme; Fig. 6a). A trial began when the central start cylinder was lifted. The mice could then freely explore the arena until a digging choice was made. Digging was defined as purposeful displacement of the shavings with paws, but not superficial sniffing. The cylinder was lowered as soon as a digging choice was made. If the choice was incorrect or if 3 min elapsed, the trial was terminated and the mouse was gently encouraged back into the start cylinder. Criterion was met when the mouse completed 8 out of 10 consecutive trials correctly.

On day 7, mice completed a recall of the initial odour discrimination to criterion again (Fig. 7a,b). The ‘recall-only' group was then returned to their homecage, while the ‘reversal' group continued within the same session to learn new odour cue–outcome contingencies. The rewarded odour was switched from anise (O1) to clove (O2). A novel odour cue was also introduced, with eucalyptus replacing thyme. Mice again were required to achieve 8 out of 10 consecutive trials correct to reach criterion. Training sessions on days 6 and 7 were typically completed within ∼1.5 h.

The ‘arena control' group was yoked to the experimental conditions of the ‘reversal' group, but without food rewards (Supplementary Fig. 1). Each day, mice entered the arena for time matched to the trained cage mate, with the same number of pots and shaving odours. However, mice were never placed in the start cylinder and there was no demarcation of trial structure. All behavioural experiments were carried out during the subjective dark period, and ‘arena control' mice actively explored the arena and shavings. Mice were not observed sleeping while in the arena. The ‘standard-housed' mice were cage mates of the ‘trained' and ‘arena control' groups, and so experienced the environment of the behaviour testing room. All groups were handled daily for weighing and imaging.

### Analysis of behavioural data

We modelled discrimination and reversal learning using a reinforcement learning model driven by an iterative error-based rule^22^,^38^,^39^. The model uses a prediction error (*δ*) to update the value (*V*) of each odour stimulus. The prediction error is the difference between the experienced feedback (*λ*) and the current expected value, where *λ* is 100 for rewarded choices and is 0 for unrewarded choices. The prediction error is scaled by a learning rate parameter (*α*), with 0<α<1.


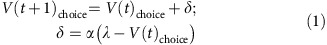


Since there may be different circuit mechanisms underlying positive and negative feedback, we also fitted behavioural data to a model with separate learning rates for rewarded (*α*_pos_) and unrewarded (*α*_neg_) outcomes^67^,^68^. When feedback is better than expected, the model generates a positive-prediction error that increases the value of that odour. Likewise, when feedback is worse than expected, the model generates a negative-prediction error that decreases the value of that odour.

Mice have innate odour preferences^69^, and the task was designed so that the rewarded odour was not the most preferred initially in discrimination. The initial values for the odours were generated from the average percentage of choice for each odour in the first four trials of the discrimination for 26 mice (for *t*=1, *V*(O1)_*t*_*=*25, *V*(O2)_*t*_*=*19, *V*(O3)_*t*_*=*5, *V*(O4)_*t*_*=*51). The initial values for the odours O1, O2 and O3 in the reversal were determined by the choice history of the animal in discrimination and recall, and the initial value of a novel odour O4′ introduced in reversal was set from the average percentage choice of that odour in the first four trials of reversal (*V*(O4′)*=*6).

To model trial-by-trial choice probabilities, the stimulus values were transformed using a softmax function to compute the relative probability of each choice. The inverse temperature parameter (*β*) determined the stochasticity of the choices.


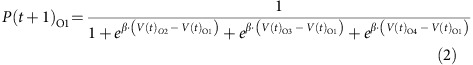


The same equation applies for all odour choice probabilities, replacing with O1, O2, O3 or O4 as appropriate. The probability (*P*) of all odour choices sums to 1.

The free parameters *α*, *α*_pos_ and *α*_neg_ and *β* were estimated separately for the discrimination and reversal training phases. The recall phase was assumed to use the same parameters as the discrimination. The best-fit parameters were optimized by maximizing the log-likelihood across trials for each animal. We compared the alternative models using the Akaike information criterion, corrected for small sample sizes (Supplementary Table 1). Model AICc values were compared using the signed-rank test, and were not found to be significantly different from the chosen models. We also tested for correlation between parameters, which would indicate that two parameters might be redundant.

For the discrimination phase, we chose the simplest model in which only *α* varied among animals, and *β* was fixed. The model with two free parameters (*α* and *β*) had a slightly lower AICc score, but the magnitude of the prediction errors was difficult to interpret since the parameters were significantly inversely correlated (Supplementary Table 1). As has been noted previously, individual parametric fits for this type of task tend to be noisy and can yield improbable results^30^,^70^. So following previous work, we fixed *β* for the group to the mean of the individual fits 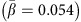. Importantly, the classification of explore and exploit trials was not significantly different between these two models for discrimination learning (explore: *t*(17)=1.23, *P*=0.24; exploit: *t*(17)=0.27, *P*=0.79). For the reversal phase, the best model used three free parameters for *α*_pos_, *α*_neg_ and *β*, and the parameters were not correlated.

We used the output of the model to classify the strategy used by the animal. Trials were classified as exploitation or exploration trials based on whether the chosen action was also the most probable choice predicted by the value-based reinforcement model^30^. Exploitation trial choice matched the most probable (and highest valued) choice. Exploration trials were choice of any of the three less probable alternative options. Omission trials were not included in the strategy classification analysis.

We also tested an alternative model using *ɛ*-greedy for action selection^22^. The algorithm selects the most valuable choice with a probability of 1–*ɛ*, and chooses randomly from the remaining options with a probability of *ɛ*/(*k*−1), where *k* is the number of options and *i* is an odour choice.


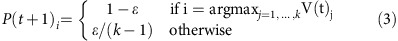


Supplementary Table 1 shows that the *ɛ*-greedy model fit the data significantly less well than the softmax model for action selection. Exploratory choices classified using the softmax model were made to the second most valuable option in 58% of explore trials, indicating that mice continued to use a value-based strategy in exploration. Exploratory trials were also more common under conditions of greater uncertainty, when the probabilities of the top two choices were more similar (mean probability difference: explore trials 0.27±0.009, exploit trials 0.38±0.02; *t*(32)=5.93, *P*<0.0001). The *ɛ*-greedy model has an equal probability for exploration of the three alternatives, and thus overestimated the probability of two of the less valuable choices. The *ɛ*-greedy model also has the same probability for exploration across all trials, and therefore poorly fit the behavioural data at the beginning and end of the session when mice were more certain of their choice.

### *In vivo* two-photon imaging of long-range axonal projections

The procedure for imaging through a chronic cranial window using two-photon imaging was performed as previously described^40^. Mice were anaesthetized with isoflurane anaesthesia, and a bar affixed to the skull was screwed into a metal post and fixed to a metal base. The brain was imaged using an Ultima IV laser scanning microscope (Prairie Technologies) and a water immersion 40x magnification 0.8 numerical aperture objective. A Mai Tai HP laser (Spectra Physics) was tuned to 910 nm for excitation of green fluorescent protein. Approximately, 80 μm segments of axon were imaged at zoom 4 with high resolution (12.05 pixels per μm) within 100 μm of the surface (layer 1). Image stacks were obtained using a 1-μm z-step. Imaging for the main experiments was within 0.8 mm of the midline in the right hemisphere, contralateral to the viral injection. A smaller set of regions of interest were studied in the ipsilateral left hemisphere (0–0.8 mm from midline), and lateral right hemisphere (0.8–1.4 mm) for comparison of density and turnover (Supplementary Fig. 2). For relocation of the same axons across imaging sessions, a bright-field image and a two-photon image stack were taken of the pattern of the blood vessels and neuronal processes as a reference point. After imaging, mice were given subcutaneous saline and allowed to recover in a separate cage before returning to the homecage.

### Bouton scoring

Boutons were scored according to established criteria^40^, using custom Matlab software (Mathworks) to measure the intensity of a varicosity relative to the axon shaft. Varicosities were scored as boutons if two or more pixels were more than three times as bright as the axon shaft within the adjacent 2 μm. A new varicosity was scored as a gained bouton if it met these same criteria. A bouton was subsequently scored as lost if it fell below 1.3 times as bright as the adjacent shaft. Varicosity peaks had to be >2 μm apart to score individually. Scoring was done based on individual *z*-sections of three-dimensional image stacks, choosing the brightest section for analysis of each varicosity. A bouton was considered to be the same across time points if it was within 1 μm of the expected position, based on distance to nearby landmarks or stable boutons. File names were recoded for analysis, so that all scoring was done blind to the experimental condition. In total, 35 mice were imaged including 323 axons and 6,512 unique boutons.

### Analysis of bouton dynamics

Bouton gain and loss were scored in comparison with the previous imaging session, as a percentage of the total boutons present that day. Turnover was the number of boutons gained and lost in a session, divided by two times the total boutons present that day. Normalized values for gain, loss and turnover were obtained by dividing the measurement by the average of the two measurements from the baseline imaging sessions, yielding a fold change relative to the baseline days. The survival fraction of boutons was calculated by starting with the total number of boutons gained in a specific session and plotting the fraction of this total that were present on subsequent days.

### Image processing

Images for data analysis were median filtered. Images for figures were median filtered, then projected in two dimensions from the three-dimensional image stack. Background processes were cropped out. There were normally several axons in an image. Finally, images were Gaussian filtered and contrasted for presentation.

### Statistics

Statistical analyses were performed using Matlab or GraphPad Prism 5. Groups were tested for normal distributions in the data. Two-way repeated measures analysis of variance was used to compare groups across imaging sessions, with Bonferroni corrected *post hoc* tests for each session. Comparisons were made among groups at a single time point using one-way analysis of variance, with Bonferroni corrected *post hoc* tests comparing the ‘arena control' group to the other experimental conditions. Comparisons between two groups were made using unpaired *t*-test, or within the same animal between two behavioural sessions using paired *t*-test. Pearson's correlation was used to measure the linear relationship between parameters. One outlier in the normalized bouton loss data from discrimination and reversal was identified using Grubb's test, and that data point was excluded from analysis. Kolmogorov–Smirnov tests were used to test for differences in cumulative histogram distributions.

## Additional information

**How to cite this article:** Johnson, C. M. *et al.* Rule learning enhances structural plasticity of long-range axons in frontal cortex. *Nat. Commun.* 7:10785 doi: 10.1038/ncomms10785 (2016).

## Supplementary Material

Supplementary Information Supplementary Figures 1-3 and Supplementary Table 1

## Figures and Tables

**Table 1:** Experimental timeline.

**Imaging day**	**Baseline imaging**			**Training (a.m.)+Imaging (p.m.)**				**Imaging**
	1	2	3	4	5	6	7	8
Standard housed	H	H	H	H	H	H	H	H
Arena control	H	H	H	Arena	Arena	Arena	Arena	H
Recall only	H	H	H	Habituation	Shape	Discrim.	Recall	H
Reversal	H	H	H	Habituation	Shape	Discrim.	Recall and Reversal	H

Discrim., discrimination; H, homecage.

Timeline of behavioural and imaging sessions for each group. ‘Untrained' mice consist of ‘standard-housed' and ‘arena control' groups. ‘Trained' mice include the ‘recall-only' and ‘reversal' groups that have identical behavioural conditions until day 7.

**Table 2:** The number of rewards or trials do not correlate with bouton plasticity.

	**Discrimination**	**Reversal**
*Total rewards*		
Bouton gain	*R*=0.16, *P*=0.54	*R*=0.21, *P*=0.58
Bouton loss	*R*=0.17, *P*=0.54	*R*=0.30, *P*=0.47
		
*Trials to criterion*		
Bouton gain	*R*=0.21, *P*=0.42	*R*=-0.02, *P*=0.96
Bouton loss	*R*=0.05, *P*=0.84	*R*=0.46, *P*=0.25

Total rewards are the number of correct trials achieved before reaching criterion of 8 out of 10 consecutive trials correct. Trials to criterion include both correct and incorrect trials. Discrimination: *n*=17 mice; reversal: *n*=9 mice. Pearson's correlation coefficient.
