# Supplementary material for: Rule learning enhances structural plasticity of long-range axons in frontal cortex
Source: Nat Commun. 2016 Mar 7;7:10785. doi: 10.1038/ncomms10785 (PMC4786641; doi:10.1038/ncomms10785)
Supplement: Supplementary Information — Supplementary Figures 1-3 and Supplementary Table 1 [file ncomms10785-s1.pdf]

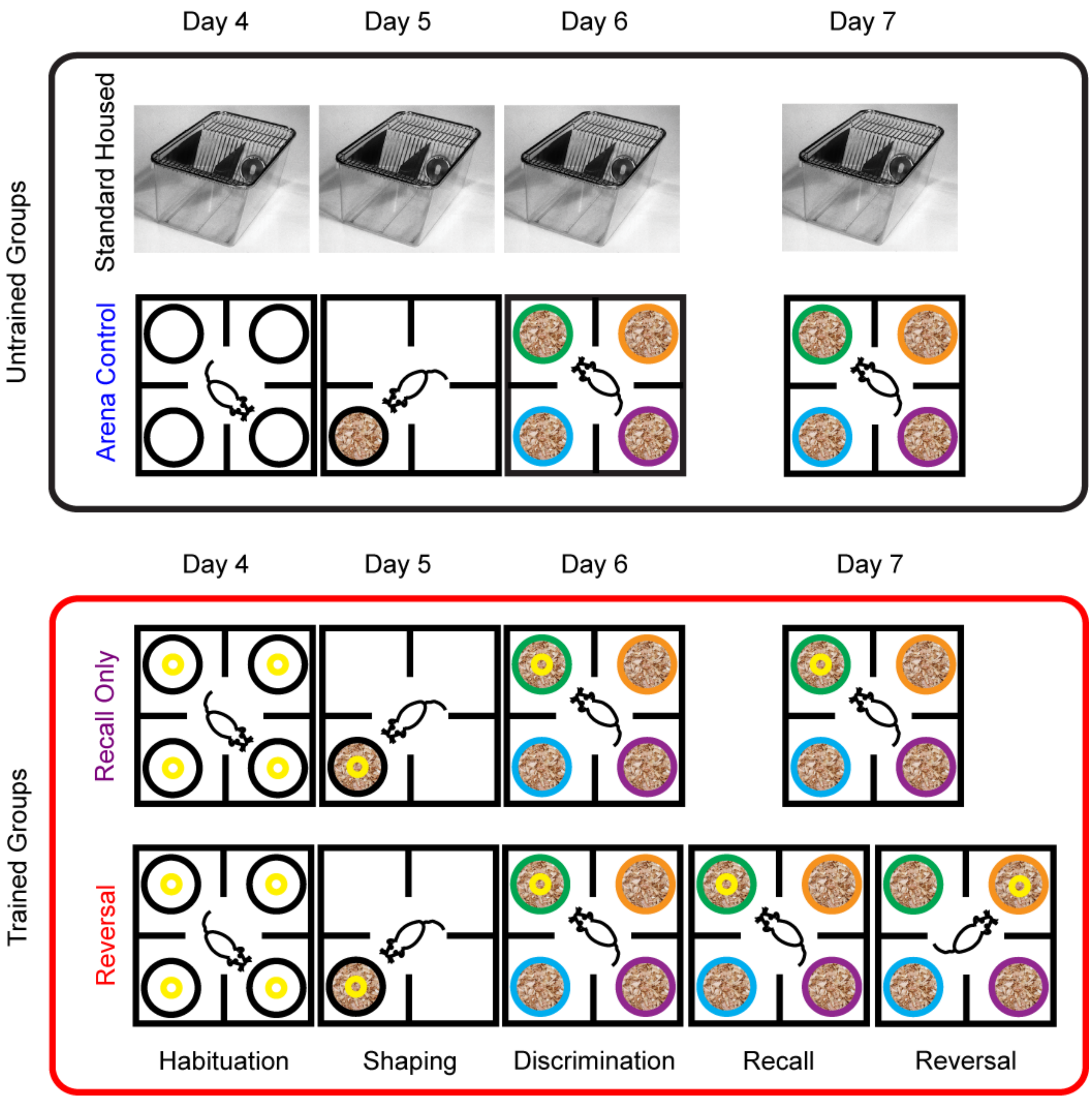

Supplementary Figure 1. Behavioral groups. Schematic of the behavioral conditions for the all the groups on days 4-7. The ‘Untrained’ groups include ‘Standard Housed’ and ‘Arena Control’ groups. ‘Standard Housed’ mice were litter mates of the other groups. The ‘Arena Control’ group experienced the same sensory cues as the trained mice, but without food rewards. There was no overt trial structure or changing of pot location in the ‘Arena Control’ sessions. The ‘Trained’ groups include the ‘Recall Only’ and ‘Reversal’ groups which only diverge on day 7. On day 7, both ‘Trained’ groups perform a recall of the odor discrimination learned on day 6. The ‘Recall Only’ group was returned to the homecage after recall and the ‘Reversal’ group went on to learn a new odor reward contingency. Yellow circles represent buried cereal rewards. All bowls were sham baited with an inaccessible piece of cereal. Color outlines of four bowls of wood shavings indicate the individual odor cues. The spatial locations of the four choices were changed on each trial.

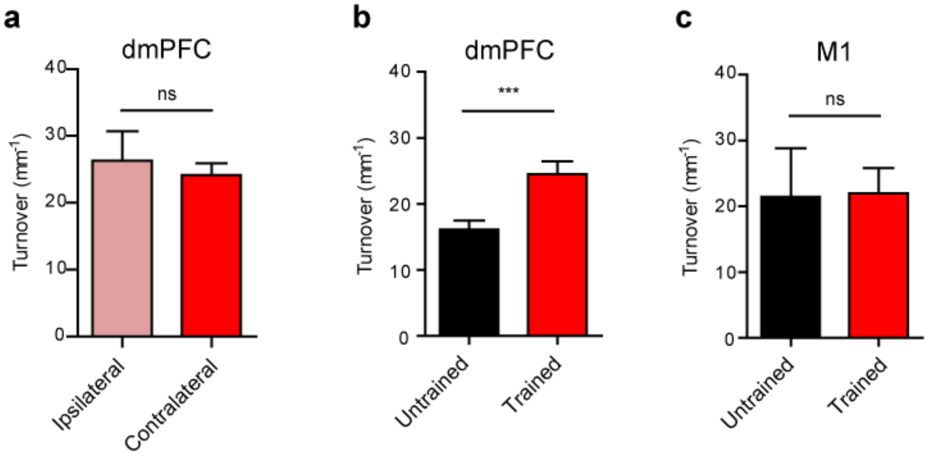

Supplementary Figure 2. Specificity of bouton turnover in OFC projection targets on day 6. a, The turnover of OFC axonal boutons was not different between ipsilateral (n=19 axons) and contralateral (n=128 axons) projections to the dmPFC following discrimination training on day 6 in ‘Trained’ mice ($t(145)=0.33$, $P=0.74$; unpaired t-test). **b**, OFC axons projecting to the dmPFC in the ‘Trained’ group (n=128 axons) had significantly higher bouton turnover than in ‘Untrained’ group (n=163 axons) ($t(289)=3.63$, $P=0.0003$; unpaired t-test). **c**, There was no difference in OFC projection to primary motor cortex (M1) bouton turnover between ‘Untrained’ (n=12 axons) and ‘Trained’ (n=20 axons) mice following discrimination training on day 6 ($t(30)=0.07$, $P=0.94$; unpaired t-test). Graphs show mean±SEM.

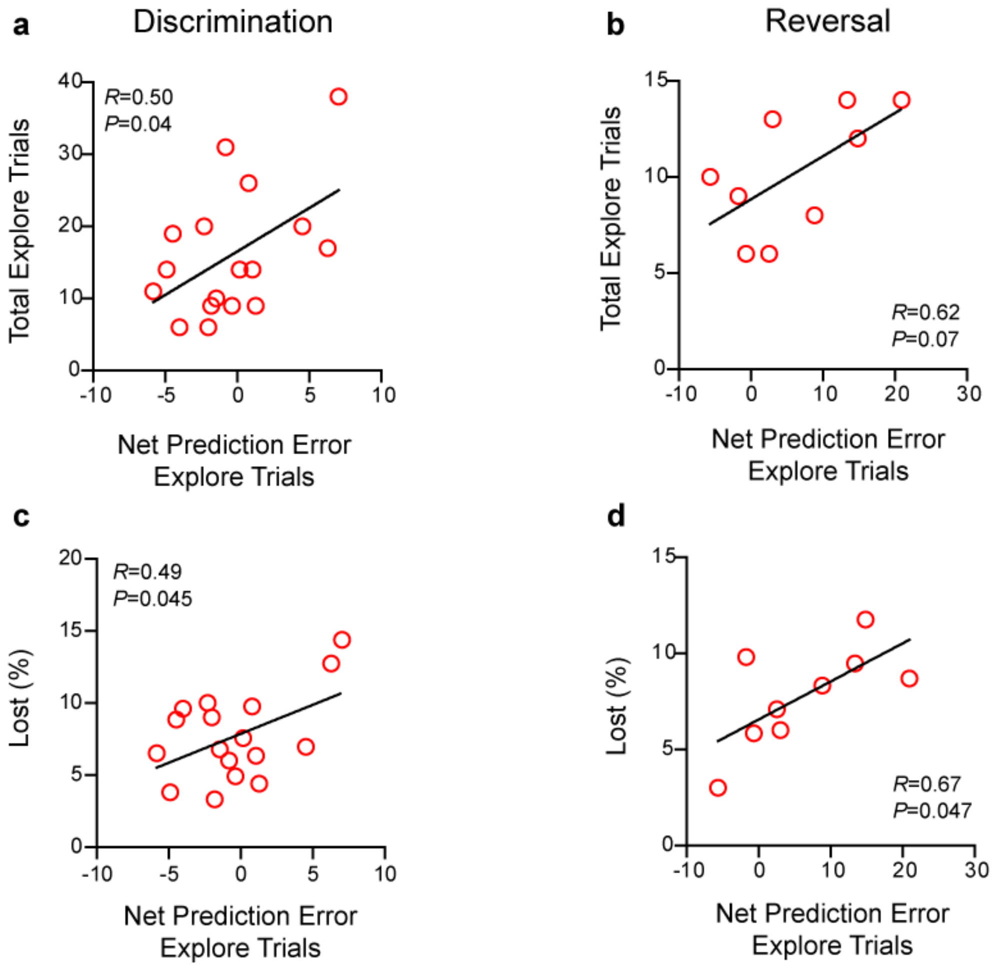

Supplementary Figure 3. Relationship between prediction errors from exploration and bouton loss. a, **b**, The total number of exploratory trials is positively related to the net prediction errors calculated from exploratory trials in discrimination training (**a**) and reversal training (**b**). Over the course of a training session, if exploration yielded better than expected outcomes, mice also made more exploratory choices. **c**, **d**, The prediction errors from exploratory trials in discrimination (**c**) and reversal (**d**) significantly correlate with the percentage of boutons lost that session. When exploratory choices outcomes were better than expected, mice also lost more boutons. Each symbol is one mouse. Pearson’s correlation coefficient.

	Parameters	AICc	Model Compare *P*-value	Parameter Correlation *P*-value
**Discrimination**				
Softmax				
α	1	1371	1	NA
α, β	2	1368	0.51	0.03
$\alpha_{pos}, \alpha_{neg}, \beta$	3	1385	0.17	α_{neg}, 0.26; β, 0.11
ℰ-greedy				
$\alpha, \mathcal{E}$	2	1437	0.01	0.32
**Reversal**				
Softmax				
α	1	554	0.91	NA
α, β	2	527	0.25	0.04
$\alpha_{pos}, \alpha_{neg}, \beta$	3	525	1	α_{neg}, 0.29; β, 0.07
ℰ-greedy				
$\alpha_{pos}, \alpha_{neg}, \mathcal{E}$	3	681	0.004	α_{neg}, 0.002; ε, 0.09

Supplementary Table 1. Model comparison. The Akaike information criterion corrected for small sample sizes (AICc) was used to compare the fit of candidate models (Softmax and ℰ-greedy) as well as the number of free parameters in these models. Models were fit separately for the discrimination and reversal phases of the task. The chosen model is shaded grey. AICc values were compared to the chosen model using the signed-rank test. The ℰ-greedy model was significantly worse than the chosen Softmax model. We also tested for correlation between parameters within a given model to test for independence of parameters.
